# Identification of the Fungal Community in Otomycosis by Internal Transcribed Spacer Sequencing

**DOI:** 10.3389/fmicb.2022.820423

**Published:** 2022-03-15

**Authors:** Xiaona Gu, Xiangrong Cheng, Jinhua Zhang, Wandong She

**Affiliations:** ^1^Nanjing Drum Tower Hospital Clinical College of Traditional Chinese and Western Medicine, Nanjing University of Chinese Medicine, Nanjing, China; ^2^Department of Otolaryngology, Affiliated Hospital of Integrated Traditional Chinese and Western Medicine, Nanjing University of Chinese Medicine, Nanjing, China; ^3^Department of Laboratory Medicine, Affiliated Hospital of Integrated Traditional Chinese and Western Medicine, Nanjing University of Chinese Medicine, Nanjing, China; ^4^Department of Otolaryngology-Head and Neck Surgery, Nanjing Drum Tower Hospital, The Affiliated Hospital of Nanjing University Medical School, Nanjing, China

**Keywords:** otomycosis, ITS sequencing, *Aspergillus*, *Malassezia*, bifonazole, fungal identification

## Abstract

We used internal transcribed spacer (ITS) sequencing to identify the fungal community in otomycosis patients and to evaluate the treatment effects of bifonazole. Ten patients who visited the Department of Otolaryngology of Jiangsu Provincial Hospital on Integration of Chinese and Western Medicine from May 2020 to April 2021 were recruited. Otomycosis patients were treated with bifonazole solution once a day for 14 days. Samples collected from the external auditory canal before and after treatment (Pre-treatment, *n* = 14 ears; Post-treatment, *n* = 14 ears) were used for microscopic examination, fungal culture, and ITS sequencing. Samples collected from 10 volunteers (Control, *n* = 20 ears) were used as controls. The symptoms, including ear itching, aural fullness, otalgia, hearing loss, and physical signs were recorded before treatment as well as on the 7th and 14th days after treatment. *Aspergillus* was identified as a main pathogenic fungus by microscopic examination, fungal culture, and ITS sequencing. At the genus level, *Aspergillus* was more abundant in the pre-treatment group than the control and post-treatment groups, and *Malassezia* was more abundant in the control and post-treatment groups than the pre-treatment group. The fungal species richness and diversity reduced significantly in the pre-treatment group compared with the control and post-treatment groups. The effective rate of bifonazole was 64.29% and 100% on the 7th and 14th days after treatment, respectively. In conclusion, the results obtained from morphologic studies and ITS sequencing indicate that *Aspergillus* is the main pathogenic fungus of otomycosis patients in Nanjing, Jiangsu Province, China. *Malassezia* is the dominant resident fungi in healthy individuals. ITS sequencing provides comprehensive information about fungal community in otomycosis and is helpful in evaluating the efficacy of antifungal agents.

## Introduction

Otomycosis, also called otitis externa mycotica, is an inflammatory lesion caused by the invasion or the excessive propagation of pathogenic fungi in the external auditory canal (Vennewald and Klemm, 2010). It is a common disease, accounting for approximately 10–20% of ear canal infections (Kiakojuri et al., 2021). Globally, otomycosis is highly prevalent in tropical and subtropical areas with high temperature and high humidity. Otomycosis is often seen in people with antibiotic and steroid use, or who have a weakened immune system or diabetes (Viswanatha and Naseeruddin, 2011; Jia et al., 2012). Other risk factors include exposure to contaminated water, frequent ear picking, and chronic otitis media (Jin et al., 2015; Chen et al., 2021). It causes symptoms such as ear itching, aural fullness, otorrhea, otalgia, hearing impairment, and tinnitus.

The *Aspergillus* species *Aspergillus niger* and *Aspergillus flavus*, followed by the *Candida* species *Candida albicans*, have been reported to be the most common pathogens of otomycosis in the literature (Fang et al., 2019; Tasić-Otašević et al., 2020). However, fungal communities in otomycosis may vary in different areas and in patients with or without underlying conditions. For example, according to studies in Iran (Sabz et al., 2019; Kiakojuri et al., 2021), *Aspergillus tubingensis* (18/45 cases), not *A. niger* (6/45), is dominant in otomycosis in western China (Zhang et al., 2020), while *Aspergillus terreus* is a dominant fungus in Hangzhou, a city in southeast China (Zhang et al., 2021). In a coastal city in India, both *A. niger* and *Aspergillus fumigatus* are dominant (Prasad et al., 2014). Apparently, *C. albicans* is often seen in immunocompromised patients (Viswanatha et al., 2012).

Early identification of fungal pathogens is critical for the diagnosis and treatment of otomycosis. Traditional methods used to identify fungal species, such as microscopic examination, isolation, and culture, are based on the morphological characteristics of fungus (Viswanatha et al., 2012). However, there are some limitations in these traditional methods, for example, staining methods, the quality of microscopes used, and the experience or subjective judgment of laboratorians. Laboratory contamination during culture is also a concern (Campbell et al., 2013; Fang et al., 2019). The traditional methods could only identify the main pathogens in most cases (Campbell et al., 2013). All of these limitations make it difficult to accurately identify fungal species by these methods (Sabz et al., 2019). Compared to ITS sequencing, only 28.57% consistent results were obtained by culture (Ren et al., 2020). In recent years, next-generation sequencing (NGS) technologies have been widely used to identify fungi in fungal infections in clinics for its objectivity and comprehensiveness. Eight causative fungal genera were identified by ITS sequencing while only five were identified by culture in fungal keratitis (Ren et al., 2020). In addition, the sequencing methods are able to detect fungi that are refractory to culture and identify some rare fungal species in the respiratory system (Charlson et al., 2012; McTaggart et al., 2019) or in fungal community studies in dermatological diseases (Woldeamanuel et al., 2006; van Diepeningen et al., 2015), as well as in catheter-related candidemia in patients with cardiovascular disorders (Hashemi Fesharaki et al., 2018). However, ITS sequencing analysis has not been widely used in fungal identification in otomycosis.

Several topical antifungals including bifonazole, clotrimazole, miconazole, and tolnaftate are effective for treatment of otomycosis and safe even in patients with tympanic membrane perforation (Vennewald and Klemm, 2010). Antifungal ear drops achieve an efficacy of higher than 80% on initial application after cleaning the external auditory canal (Jia et al., 2012).

In the present study, we used microscopy, culture, and ITS sequencing analysis to identify the pathogen of otomycosis and evaluate the treatment effects of bifonazole on the fungal community in the external auditory canal in the city of Nanjing of China.

## Materials and Methods

### Ethics and Consent

Otomycosis patients were recruited from the Department of Otolaryngology, Jiangsu Provincial Hospital on Integration of Chinese and Western Medicine from May 2020 to April 2021. This study was approved by the Committee of Jiangsu Provincial Hospital on Integration of Chinese and Western Medicine (No: 2020LWKY001). Written informed consent was obtained from each patient and volunteer who participated in this study.

### Groups, Treatment, Clinical Symptoms, and Sign Observation

Ten otomycosis patients (14 ears) who met the inclusion criteria were recruited in the present study. Diagnosed patients were treated with 1% bifonazole solution (H20066175; Sinomune Pharmaceutical Co., Ltd, Wuxi, China.) once a day for 14 days. Fiberoptic otoscopy was performed before and after cleaning the external auditory canal on the first visit, as well as on the 7th and 14th days of treatment. Clinical symptoms including ear itching, aural fullness, otalgia, hearing loss, and physical signs were recorded before and after bifonazole treatment (Table 1).

**TABLE 1:** Symptom and physical sign rating scales.

Primary symptoms	Score
**Ear itching**	
None	0
Mild itching that is often ignored	3
Moderate itching that can be relieved by scratching the ear canal and does not affect sleep	6
Unbearable severe itching that cannot be relieved by scratching the ear canal and affects asleep	9
**Aural fullness**	
None	0
Mild ear tightness that is often ignored	2
Ear distension that affects work and sleep slightly	4
Unbearable ear distension that affects work and sleep seriously	6
**Secondary symptoms**	
**Otalgia**	
None	0
Slight earache that is often ignored	1
Frequent earache that does not affect work and sleep	2
Unbearable earache that affects work and sleep	3
**Hearing loss**	
None	0
Slight hearing loss that is often ignored	1
Conscious hearing loss that does not affect daily communication	2
Significant hearing loss that affects daily communication	3
**Physical signs of the external auditory canal**	
The external auditory canal is clean and the tympanic membrane is distinct	0
A little discharge in the external auditory canal with a visible tympanic membrane	3
The external auditory canal is wet with fungal mycelium-like discharge, but does not exceed half the volume of the ear canal, and the tympanic membrane is not visible	6
The external auditory canal is clogged with fungal mycelium-like discharge, and the tympanic membrane is not visible	9
**Standard for evaluation of curative effect**	
Recovery	The total score of symptoms and signs decreased by >95%
Significantly effective	The total score of symptoms and signs decreased by 70–95%
Effective	The total score of symptoms and signs decreased by 30–69%
Ineffective	The total score of symptoms and signs decreased by <30%.

The medical history of each patient, such as exposure to contaminated water or ear picking, was recorded. Other related diseases such as chronic otitis media, diabetes, other fungal infections, family history of fungal infections, and antibiotic use were also recorded.

The 10 patients in the present study, five men and five women, were aged from 21 to 55 years. Six cases were unilateral and four were bilateral (totaling 14 ears) with a course of 1 week to 4 years. All of them had a history of ear picking, four had a history of swimming in polluted water, four had other fungal infections such as tinea pedis or tinea cruris, and five had a family history of fungal infections (tinea pedis or tinea cruris). None of them had a history of chronic otitis media, diabetes, or antibiotic use.

Ten healthy volunteers (20 ears) were recruited as the control group. Samples from both patients (Pre-treatment and Post-treatment) and volunteers were collected for subsequent studies.

### Sampling

Sterile sampling swabs soaked with sterile normal saline were used to collect the fungal mycelium-like discharge and stored in sterile sampling tubes for smear, microscopic examination, or culture. Two or three sampling swabs from each patient or volunteer were sealed into a sterile cryogenic vial and stored at −80°C for DNA isolation and polymerase chain reaction (PCR) amplification.

### Microscopic Examination and Fungal Culture

The discharge collected from the ear canal was evenly smeared onto glass slides. The slides were then air-dried, stained with Gram staining solution, and examined with a biological microscope (CX23LEDRFS1C; Olympus, Japan) by using 40 × and 100 × oil-immersion objectives. The specimens for fungal culture were inoculated on Sabouraud Agar plates (90 mm, Autobio Diagnostics Co., Ltd., Zhengzhou, China) by streaking and cultured at 28°C in an incubator for 2–4 days. The cultured fungus was collected, smeared on slides, stained with lactophenol cotton blue, and examined *via* microscope. The cellophane tape method was used in smearing (Campbell et al., 2013).

### DNA Extraction and PCR Amplification

A total of 48 samples collected from patients and normal controls were used for ITS sequencing. Fungal DNA was extracted using an E.Z.N.A.^®^ Fungal DNA Kit (D3390-00; Omega Bio-Tek, Norcross, GA, United States) according to the manufacturer’s protocol. More than 500 ng of DNA in a concentration of 10 ng/μl or higher was extracted from each sample.

The ITS1F-ITS2R region of the fungal ITS gene was amplified by PCR amplifier (GeneAmp PCR System 9700; Applied Biosystems, Foster City, CA, United States) using ITS primers (ITS 1F 5′-CTTGGTCATTTAGAGGAAGTAA-3′; ITS 2R 5′-GCTGCGTTCTTCATCGATGC-3′). Triplicate PCR reactions were performed in a 20 μl mixture containing 4 μl of 5 × FastPfu Buffer, 2 μl of 2.5 mM dNTPs, 0.8 μl of each primer (5 μM), 0.4 μl of FastPfu Polymerase (TransStart, AP221-13; TransGen Biotech Co., Ltd., Beijing, China), and 10 ng of template DNA. The thermal cycle conditions of PCR included a 5-min initial denaturing at 95°C, followed by 29 cycles of 30-s denaturing at 95°C, 30-s annealing at 55°C and 45-s extension at 72°C, plus a final extension at 72°C for 10 min.

The amplicons were qualified by 2% agarose gel electrophoresis at 220 V and 90 mA for 45 min and purified using the AxyPrep DNA Gel Extraction Kit (Axygen Biosciences, Union City, CA, United States) according to the manufacturer’s instructions.

### Library Construction and Sequencing

After quantification by Qubit^®^ 3.0 (Life Invitrogen, Carlsbad, CA, United States), the PCR products were pooled equally and used for library construction. Pair-end library was prepared using Illumina TruSeq DNA PCR-Free Library Preparation Kit (20015963; Illumina, San Diego, CA, United States) and Illumina’s genomic DNA library preparation procedure. The library was then sequenced on an Illumina platform (Shanghai Biozeron Biotech. Co., Ltd., Shanghai, China) using the paired-end 2 × 250 bp method according to the standard protocol.

### Data Processing

The low-quality raw fungal sequences with an average quality score < 20 bp and reading shorter than 50 bp by QIIME (v.1.17) were discarded. The retained sequences were clustered into operational taxonomic units (OTUs) with 97% similarity cutoff using UPARSE^1^ (v.7.1). The taxonomic analysis on OTUs was identified by RDP Classifier^2^ and a Bayesian algorithm in the UNITE database^3^ (Release 8.2) with a confidence threshold of 0.7.

### Alpha- and Beta-Diversity Analyses

The rarefaction analysis based on Mothur (v.1.21.1) was conducted to reveal the α-diversity indices, including the coverage, richness (Chao1), and diversity (Shannon) indices (Schloss et al., 2009). UniFrac was used for β-diversity analysis (Lozupone et al., 2011). Principal coordinate analysis (PCoA) and analysis of similarities (ANOSIM) based on Bray-Curtis similarity were used to evaluate the statistical difference in diversity index between samples. Kruskal–Wallis rank-sum test was used to analyze the changes and differences between samples. *p* < 0.05 was considered to be statistically significant.

### Linear Discriminant Analysis Effect Size Analysis

Linear discriminant analysis effect size analysis was conducted to identify biomarkers of fungi (Segata et al., 2011). Kruskal–Wallis rank-sum test was performed for identification, followed by LDA analysis to determine each distinctively abundant taxa (Ijaz et al., 2018).

### Statistical Analysis

All statistical analyses were performed using SPSS 23.0 (SPSS Inc., Chicago, IL, United States). Measurement data with normal distribution was expressed as mean ± SD and analyzed by Student’s *t*-test, and Mann–Whitney *U* test was used for data with abnormal distribution. Enumeration data were expressed as rate (%) and analyzed by chi-square test. *p* < 0.05 was considered statistically significant.

## Results

### Comparison of Symptom and Sign Scores, and Efficacy of Bifonazole Treatment

**FIGURE 1:**
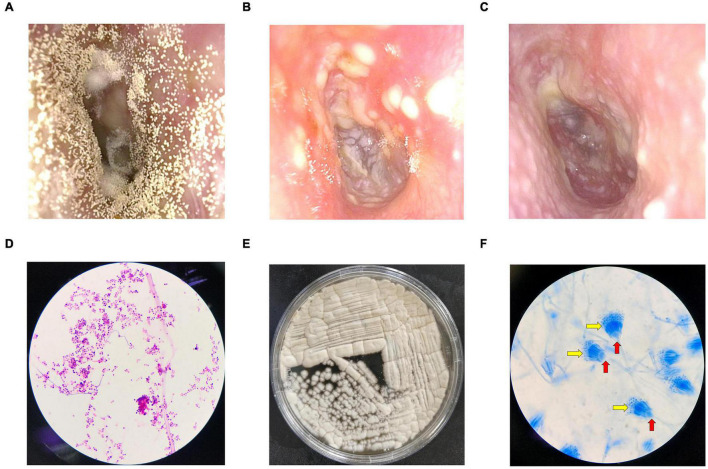
Fiberoptic otoscopy, fungal culture, and microscope examination. **(A)** An example of the external auditory canal before treatment. **(B)** Seven days after bifonazole treatment. **(C)** Fourteen days after bifonazole treatment. **(D)** Gram-stained smear at 100 × magnification. **(E)** An example of fungal culture. **(F)** Lactophenol cotton blue stained smear at 100 × magnification. Red arrows refer to conidiophore vesicles, and yellow arrows refer to fan-shaped head.

Symptoms such as ear itching, aural fullness, and hearing loss were significantly relieved both on the 7th and 14th days after treatment (*p* < 0.05 or *p* < 0.01) with improved physical signs (*p* < 0.01; *p* < 0.01, Table 2). The total scores of the post-treatment group were significantly lower than those of the pre-treatment group (*p* < 0.01, Supplementary Material). Total effective rate reached 100% after 14 days of treatment, while it was only 64.29% on Day 7 (Table 2). The discharge in the external auditory canal observed before treatment (Figure 1A) decreased (Figure 1B) and later disappeared (Figure 1C) after treatment.

**TABLE 2:** Comparison of symptom and sign scores, effective rates in the pre- and post-treatment groups.

Symptoms or Signs	Scores*			*Z*_1_ **	*p*_1_	*Z_2_/t*	*p*_2_
	
	Pre-treatment	Post-treatment					
		
		7th day	14th day				
Ear itching	5.57	4.29	1.93	−2.449	0.014	−3.169	0.002
Aural fullness	3.29	2.14	0.57	−2.828	0.005	−3.071	0.002
Otalgia	0.29	0.07	0.00	−1.732	0.083	−1.633	0.102
Hearing loss	0.93	0.29	0.00	−3.000	0.003	−2.739	0.006
Physical sign	6.21	3.64	0.86	−3.464	0.001	−3.494	0.000
Total score	16.29	10.43	3.36	−3.321	0.001	13.419***	0.000
Effective rate (n, %)	–	9 (64.29%)	14 (100%)	–	–	–	–
Cured	–	0	4				
Markedly effective	–	0	7				
Effective	–	9	3				
No response	–	5	0				

*Data with skewed distribution are expressed as average; non-parametric test was used.

**Z_1_ and p_1_ indicate comparison between pre-treatment and 7 days after treatment, while Z_2_ and p_2_ indicate comparison between pre-treatment and 14 days after treatment.

***Data (total score) with normal distribution.

### Microscopic Examination and Fungal Culture

Scattered fungal hyphae or spores were observed in all 14 samples from the pre-treatment group (Figure 1D), and 12 of them produced sandy-colored or gray-yellow granular colonies after culture (Figure 1E). Typical conidiophore vesicles and fan-shaped heads were observed with cotton blue staining (Figure 1F), indicating the reproduction of *Aspergillus*. Fungal culture (Figure 1E) and the cotton blue staining results suggest *Aspergillus terreus* (Campbell et al., 2013). No hyphae or spores were observed in the control group. No fungal colony grew in culture in the control group.

### Data Processing

After quality filtering and removal of chimeric sequences, a total of 2,591,065 sequences were yielded, with an average output of 47,798 sequences and an average length of 266 bp. More than 99.97% of the Good’s coverage was estimated in each group, indicating sufficient sequencing depth for the fungal diversity study (Table 3).

**TABLE 3:** ITS sequencing parameters of three groups.

	Control	Pre-treatment	Post-treatment
Observed OTUs	74.34 ± 18.66^a^	50.36 ± 24.51^b^	85.77 ± 26.79^a^
Chao1	85.87 ± 19.77^ab^	69.29 ± 25.74^b^	99.42 ± 26.92^a^
Good’s coverage	0.9998 ± 0.0001^a^	0.9997 ± 0.0001^a^	0.9997 ± 0.0001^a^
Shannon	1.832 ± 0.6444^ab^	1.060 ± 1.192^b^	2.225 ± 1.099^a^
Simpson	0.5096 ± 0.1760^a^	0.2568 ± 0.2553^b^	0.5701 ± 0.2372^a^

*OTU, operational taxonomic unit; ITS, internal transcribed spacer. No significant difference (p < 0.05) if two groups sharing a same superscript letter (a or b) in a sequencing parameter.*

Sequences with similarity ≥ 97% between reads were clustered into one OTU. A total of 3,933 OTUs were observed in three groups. The observed number of OTUs in the pre-treatment group was significantly lower compared to the control and post-treatment groups (all *p* < 0.05; Table 3). Moreover, the Chao1, Shannon, and Simpson indices were significantly lower in the pre-treatment group compared to the post-treatment group (all *p* < 0.05; Table 3). These results indicate a reduction in the richness and diversity of fungal species in otomycosis.

### Principal Coordinates Analysis

**FIGURE 2:**
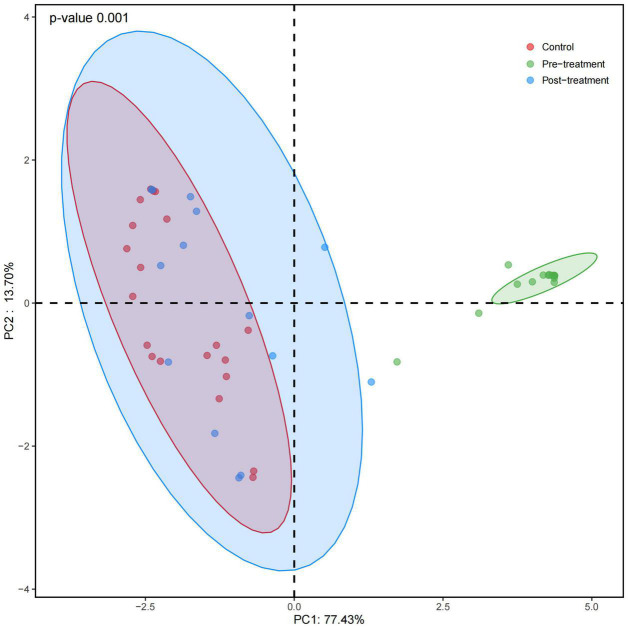
Principal coordinates analysis (PCoA) of fungal composition based on weighted UniFrac in three groups. Red dots refer to the control group. Green and blue dots refer to the pre- and post-treatment groups, respectively. Principal coordinates 1 and 2 explain 77.43% and 13.70% of the variation. PCoA of Bray-Curtis distance indicates no effective discrimination between the control and post-treatment groups. In contrast, the pre-treatment group is discriminated against the control and post-treatment groups.

Weighted UniFrac distance-based fungal structure analysis was performed to evaluate differences in the fungal community compositions of three groups. PCoA of Bray-Curtis distance indicated no effective discrimination between the control and post-treatment groups (Figure 2). In contrast, the samples in the pre-treatment group were discriminated against those in the control and post-treatment groups. Principal coordinates 1 and 2 explained 77.43% and 13.70% of the variation. These results indicate a different fungal community in the pre-treatment group compared to the control and post-treatment groups.

### Taxonomic Composition of Fungal Communities by ITS Sequencing

**FIGURE 3:**
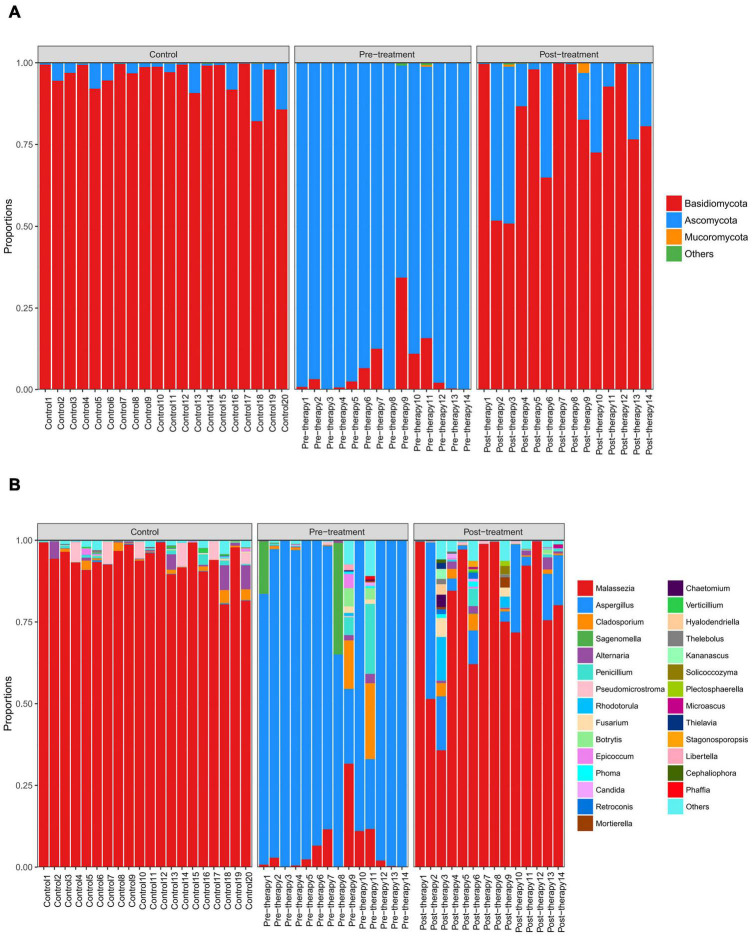
Distribution of predominant fungi in three groups at the phylum and genus levels. Different colors indicate different phylum and genus names of fungus, and the colored blocks on the right are sorted in order of abundance from high to low. **(A)** The distribution of predominant fungi at the phylum level. Basidiomycota is dominant in the control and post-treatment groups, while Ascomycota is dominant in the pre-treatment group. **(B)** The distribution of predominant fungi at the genus level. *Malassezia* is dominant in the control and post-treatment groups, while *Aspergillus* dominates in the pre-treatment group.

Two dominant fungal phyla, Basidiomycota, and Ascomycota, were observed (abundance > 1%) in all samples (Figure 3A and Table 4). Obviously, Basidiomycota was more abundant in the control and post-treatment groups than the pre-treatment group. Ascomycota was significantly increased in the pre-treatment group.

**TABLE 4:** Main taxonomic composition (>1% and the top five) of fungal communities in three groups.

Level	Control	Pre-treatment	Post-treatment
Phyla	Basidiomycota (95.70%) Ascomycota (4.19%)	Ascomycota (93.41%) Basidiomycota (6.43%)	Basidiomycota (82.58%) Ascomycota (17.05%)
Genus	*Malassezia* (93.49%) *Pseudomicrostroma* (1.76%) *Alternaria* (1.43%)	*Aspergillus* (81.92%) *Malassezia* (5.83%) *Sagenomella* (3.58%) *Cladosporium* (2.87%) *Penicillium* (2.00%)	*Malassezia* (80.26%) *Aspergillus* (10.14%) *Rhodotorula* (1.21%) *Cladosporium* (1.11%)

*The percentages in brackets indicate the abundance of species.*

At the genus level, *Aspergillus* was more abundant in the pre-treatment group compared to the control and post-treatment groups, confirming that *Aspergillus* was the dominant species in otomycosis in the present study. In addition, we detected relatively high abundance of *Sagenomella*, *Cladosporium*, and *Penicillium* in the pre-treatment group (Figure 3B and Table 4). The abundance of *Aspergillus* was ≥80% in 11 samples and >20% in three samples in the pre-treatment group. *Malassezia* was more abundant in the control and post-treatment groups compared to the pre-treatment group. The abundance of *Malassezia* was ≥80% in all 20 samples in the control group, ≥80% in eight samples, and >20% in four samples in the post-treatment group. We also detected low abundance of *Aspergillus* in the post-treatment group.

### Fungi With Significant Differences in Groups

**FIGURE 4:**
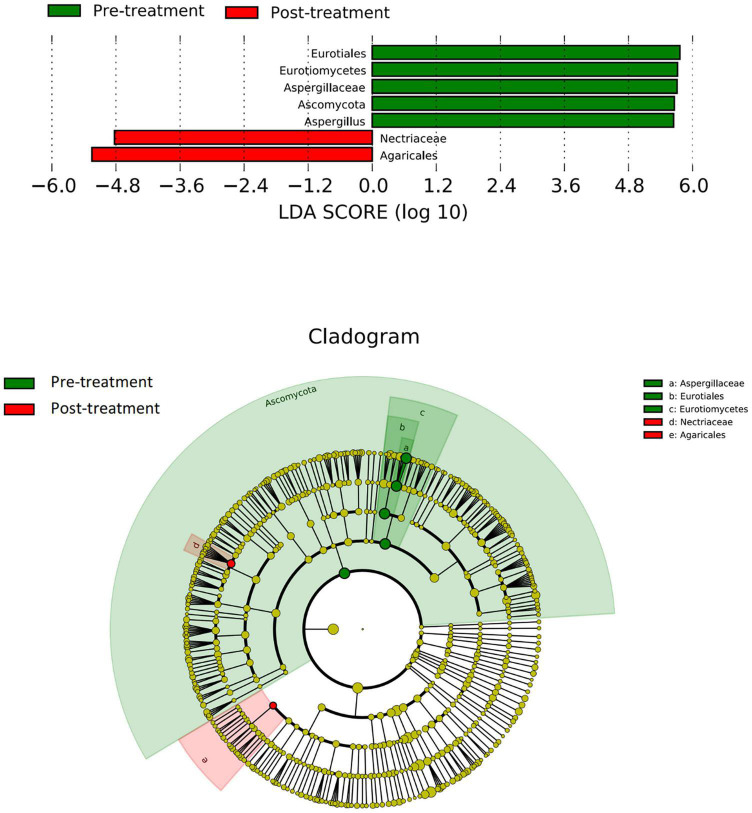
LEfSe analysis of fungal species. The bar graph shows the LDA scores calculated for characteristics at the OTU level. Green bars refer to the pre-treatment group and red ones refer to the post-treatment group. The cladogram shows the relative abundance of OTUs. Green (light and dark green, dark green caused by overlapping of light green) and pink areas represent the pre- and post-treatment groups, respectively. Green and red nodes in the branches represent fungal species that play an important role in the two groups, respectively. Yellow nodes represent fungal species that do not play an important role in both groups. The alphabetic species names are shown in the legend on the right. Eurotiales, Eurotiomycetes, Aspergillaceae, Ascomycota, and *Aspergillus* were essential fungi in the pre-treatment group while Nectriaceae and Agaricales were enriched in the post-treatment group.

There were significant differences in the community compositions between the pre- and post-treatment samples (Figure 4). Eurotiales, Eurotiomycetes, Aspergillaceae, Ascomycota, and *Aspergillus* were essential fungi in the pre-treatment group while Nectriaceae and Agaricales were enriched in the post-treatment group.

### Correlation of Fungal Genera With Symptoms and Signs

**FIGURE 5:**
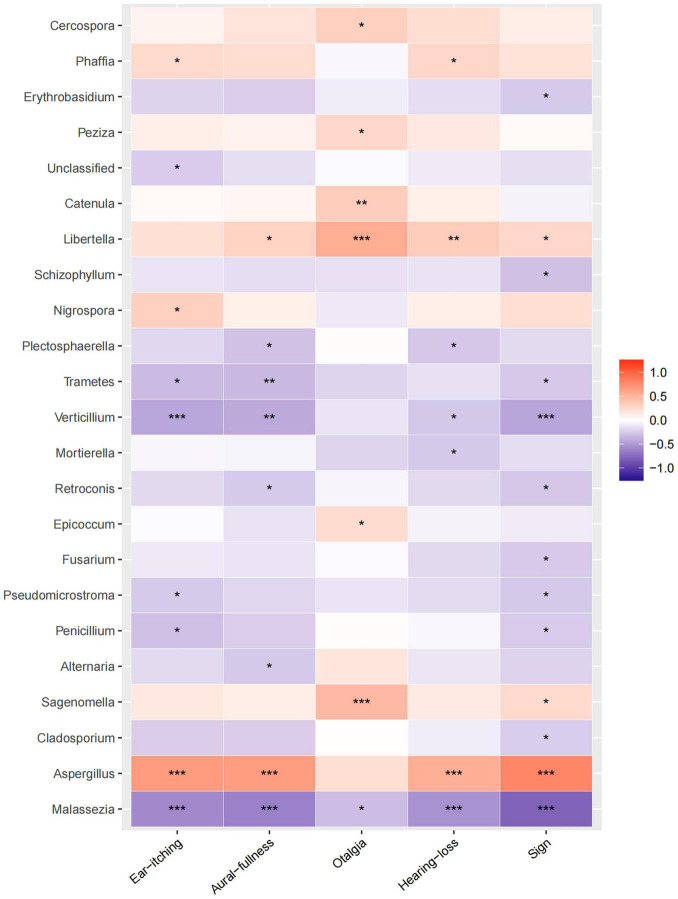
Correlation analysis between fungal abundances and clinical factors. Relative heatmap analysis between fungal genera and clinical symptoms/signs. The colors range from blue (negative correlation) to red (positive correlation). Significant correlations are noted by *p < 0.05, ^**^p < 0.01, and ^***^p < 0.001.

Relative heatmap analysis between fungal genera and symptoms/signs indicated that *Aspergillus* was associated with ear itching, aural fullness, hearing loss, and physical signs, while *Sagenomella* and *Libertella* were associated with otalgia (Figure 5). *Malassezia* was negatively correlated with ear itching, aural fullness, hearing loss, and signs, while *Vertcillium*, with ear itching and physical signs (Figure 5).

## Discussion

In the present study, scattered hyphae and spores were observed in all 14 samples collected from patients in the pre-treatment group. However, fungal species could not be identified due to structural damage caused by sampling. Using the cellophane tape method, the typical conidiophore vesicle and fan-shaped head of *Aspergillus* were observed in 12 samples. According to the color and appearance of the colonies after culture, it could be preliminarily identified as *Aspergillus* terreus. No distinct colonies were produced in culture in two samples, possibly due to low abundant or low activities of experimental strains. Therefore, *Aspergillus* can be identified by traditional methods in most cases in the present study (Table 5). However, culture-negative strains or resident fungus, such as *Malassezia* in the control group, could be hardly detected by regular culture conditions (Campbell et al., 2013). It has been reported that traditional culture-based methods have not completely defined the microbial landscape of common recalcitrant human fungal skin diseases (Findley et al., 2013). If rare and insidious strains were encountered in clinical practice, negative culture results might lead to misdiagnosis or missed diagnosis, or even delayed treatment. Fortunately, modern sequencing technologies help us solve this problem. Neji et al. (2013) and Aboutalebian et al. (2020) sequenced rare fungal pathogens (*Talaromyces purpurogenus*, *Naganishia albida*, *Filobasidium magnum*, and the Graphium stage of *Pseudallescheria apiosperma*) in otomycosis and conducted drug susceptibility tests on the isolated strains, and finally achieved successful treatment results by a combination of antifungals and corticosteroids in patients. In the present study, *Aspergillus* was detected by sequencing in two samples with negative culture results. Beside *Aspergillus*, relatively high abundance of *Sagenomella*, *Cladosporium*, and *Penicillium* were also detected by sequencing in the present study. Previous and the present studies have shown that ITS sequencing could provide important assistance in the identification of pathogenic fungi in otomycosis, especially when microscopic examination and fungal culture did not support the diagnosis.

**TABLE 5:** Comparison of different methods for fungal identification in the present study.

Method	Control (*n* = 20 ears)	Pre-treatment (*n* = 14 ears)
Symptoms and signs	No	Yes
Microscopic examination	No fungal hyphae or spores were observed	Scattered fungal hyphae and spores were observed in all 14 samples; Fungal species could not be identified
Fungal culture	No colonies grew	Colonies grew in 12 of 14 samples; Fungal species could not be identified
Microscopic examination again after culture	No fungi were observed	Typical conidiophore vesicles and fan-shaped heads were seen in 12 samples, suggesting *Aspergillus* (12/14)
ITS sequencing	A dominant fungus *Malassezia* was identified (20/20)	A dominant fungus *Aspergillus* was identified (14/14)

*The diagnosis was confirmed only by fungal hyphae and spores under microscope in patients with clinical symptoms and signs. The numbers in brackets represent the number of positive samples out of the total number of samples in each group.*

In the present study, we conducted a systematical study of fungal composition using ITS sequencing both in healthy volunteers and otomycosis patients before and after bifonazole treatment for the first time. Some major findings were achieved by this sequencing method in the present study: (1) *Aspergillus* is a main pathogen of otomycosis. (2) Other uncommon pathogens, such as *Sagenomella*, *Cladosporium* and *Penicillium*, were also detected by ITS sequencing. (3) *Malassezia* is a dominant resident fungus in the healthy external auditory canal. (4) The fungal diversity decreased significantly in otomycosis patients, indicating the occurrence of otomycosis.

Consistent with our results, *Aspergillus* is the most common pathogen in otomycosis (van Diepeningen et al., 2015; Hagiwara et al., 2019; Sabz et al., 2019), while *Malassezia* is the dominant genus in the healthy individual (Liu et al., 2020) in the literature. Very high abundance of *Aspergillus* (>80%) was detected in majority of samples in the pre-treatment group in the present study. *Malassezia* became a dominant genus in the post-treatment group, indicating recovery of fungal community after treatment. Another common genus usually detected in otomycosis, *Candida* (Tasić-Otašević et al., 2020), is not a predominant fungus found in the present study. We detected *Candida* only in seven samples with an abundance of less than 1% by sequencing. *Candida* infection is usually seen in immunocompromised patients (Viswanatha et al., 2012). No patient in the present study was immunocompromised or had a history of steroid or antibiotic use.

By ITS sequencing, we also detected *Sagenomella* and *Cladosporium* in otomycosis for the first time. The abundance of *Sagenomella* and *Cladosporium* was quite high (16.03–34.12% and 14.85–23.23%, respectively) in some cases in the present study. These two genera had not been previously reported in otomycosis. *Sagenomella* was reported only in a case of juvenile arthritis (Ried and Fakler, 2018). *Cladosporium*, a common environmental mold, was reported to be a pathogen of onychomycosis (Motamedi et al., 2016). Another genus we detected by sequencing is *Penicillium*, which was reported in otomycosis in the literature (Prasad et al., 2014). These genera are not easily found by traditional methods. *Sagenomella* and *Penicillium* are sensitive to broad-spectrum antifungal agents while antifungal treatment of *Cladosporium* remains a matter of debate (Cheng et al., 2015). Therefore, accurate identification for fungi of otomycosis is strongly desired.

The genus *Malassezia*, a group of superficial fungi as normal skin flora on the human body in the areas where sebaceous glands are rich (Findley et al., 2013), is identified as dominant fungi in the control and post-treatment samples in the present study. The abundance of *Malassezia* was significantly reduced in otomycosis patients. Although *Malassezia* is a normal residence in the auditory canal, it could be invasive and cause opportunistic infection in otomycosis (Latha et al., 2010). Special treatments are needed in that case. Therefore, comprehensive pathogen identification should be performed before treating otomycosis. High abundance of *Malassezia* (>30%) was observed in one case in the pre-treatment group in the present study.

Changes in OTU abundances could be observed in various diseases, including otomycosis (Ijaz et al., 2018; Liu et al., 2020). In the present study, the OTUs significantly decreased in the pre-treatment samples compared to the control samples (*p* < 0.05; Table 3) and increased to the control levels after treatment (*P* > 0.05; Table 3). These results indicate the following points: (1) In the control group, the levels of resident fungal floras were low and the diversity was high in the external auditory canal. (2) In otomycosis patients, the overgrowth of *Aspergillus* broke the balance of the fungal community in the auditory canal, inhibited the growth of normal fungus flora, and reduced the diversity. The overgrown fungal structures blocked the external auditory canal (Figure 1A), which affected the ventilation and drainage, then resulted in symptoms and signs. (3) Bifonazole inhibited the overgrowth of *Aspergillus*, then increased the abundance of normal fungi in the ear canal (Figure 3) and restored the diversity gradually during 2 weeks of treatment (Table 3). However, bifonazole is a broad-spectrum antifungal agent. Overuse of bifonazole should be avoided. All patients treated in the present study recovered without any side effects, indicating 2 weeks of bifonazole treatment is safe and sufficient for treating otomycosis. In addition to antifungal agents, other treatment strategies targeting microbial imbalances in the auditory canal also need to be developed in the future (Findley et al., 2013).

Additionally, the visible discharge in the external auditory canal decreased gradually, and most clinical symptoms and signs were relieved within the course of treatment in the present study. However, otalgia was not significantly relieved after 2 weeks of treatment (*p* > 0.05; Table 2), indicating that otalgia might not be a typical and specific symptom of otomycosis caused by *Aspergillus*. Otalgia could be a sign of a bacterial co-infection in otomycosis patients (Enoz et al., 2009; Liu et al., 2020; Hwa and Brant, 2021). Another explanation is that the pathological changes in the auditory canal caused by the infection were not totally recovered by the end of treatment. Therefore, a longer observation may be needed in the future. Previously, the effects of antifungal agents are studied mostly by observing clinical symptoms and signs (Lee et al., 2021; Zhang et al., 2021). The present study evaluated the efficacy of bifonazole not only through clinical symptoms and signs but also fungal community detection. This strategy provides a new way to assess the treatment efficacy of antifungal drugs by examining fungal flora returning to the normal levels in the external auditory canal.

A surprising finding was that four of 10 patients in the present study had tinea pedis, and three had family members with tinea pedis. A similar finding was reported in the literature although the connection between otomycosis and tinea pedis is still not clear (Wu and Liu, 2007). Microbial communities on the feet tend to be unstable, which provide opportunities for harmful microbes to flourish (Findley et al., 2013). All 10 patients had a history of ear picking. Pathogenic fungi colonize on damaged skin, i.e., by ear picking, faster than on undamaged skin (Shen et al., 2019). We therefore hypothesize that pathogenic fungi, i.e., from tinea pedis, may easily colonize into the auditory canal with damaged skin, i.e., caused by ear picking, thus resulting in otomycosis. However, this hypothesis should be tested by examining the fungi on the feet and in the ear canal of otomycosis patients with tinea pedis in the future.

## Conclusion

In the present study, we used microscopic examination, fungal culture, and ITS sequencing to identify the fungal community in otomycosis patients in the city of Nanjing, Jiangsu Province, of the People’s Republic of China. The results obtained from morphologic studies and ITS sequencing indicate that *Aspergillus* is the main pathogenic fungus in otomycosis patients. *Malassezia* is the dominant resident fungi in the healthy external auditory canal. The fungal richness and diversity decreased significantly in otomycosis patients, indicating the occurrence of otomycosis, and increased significantly after bifonazole treatment. Although it has limitations in fungal identification at the species level, ITS sequencing provides comprehensive information about fungal community in otomycosis and is helpful in evaluating the efficacy of antifungal agents. Quantitative polymerase chain reaction (Q-PCR), metagenomic sequencing, or third-generation sequencing technology should be used for more accurate identification of pathogenic fungi in otomycosis in the future.

## Data Availability Statement

The datasets presented in this study can be found in online repositories. The names of the repository/repositories and accession number(s) can be found below: https://www.ncbi.nlm.nih.gov/, SRP331309.

## Ethics Statement

The studies involving human participants were reviewed and approved by the Committee of Jiangsu Provincial Hospital on Integration of Chinese and Western Medicine (No: 2020LWKY001). The patients/participants provided their written informed consent to participate in this study.

## Author Contributions

WS contributed to the conception and design of the study. XG and XC recruited volunteers and collected samples. XG and JZ performed the experiments. XG processed the data, performed the statistical analysis, and wrote the first draft of the manuscript. All authors contributed to manuscript revision and read and approved the submitted version.

## Conflict of Interest

The authors declare that the research was conducted in the absence of any commercial or financial relationships that could be construed as a potential conflict of interest.

## Publisher’s Note

All claims expressed in this article are solely those of the authors and do not necessarily represent those of their affiliated organizations, or those of the publisher, the editors and the reviewers. Any product that may be evaluated in this article, or claim that may be made by its manufacturer, is not guaranteed or endorsed by the publisher.

## Footnotes

1
http://drive5.com/uparse/

2
http://rdp.cme.msu.edu/

3
http://unite.ut.ee/index.php
